# Clinical Evaluation of Various Thyroid Hormones on Thyroid Function

**DOI:** 10.1155/2014/618572

**Published:** 2014-12-08

**Authors:** Hong Li, Xiaolan Yuan, Lu Liu, Jiaojiao Zhou, Chunyan Li, Peng Yang, Le Bu, Manna Zhang, Shen Qu

**Affiliations:** Department of Endocrinology, Shanghai Tenth People's Hospital, . Tongji University School of Medicine, 301 Yanchang Middle Road, Shanghai 200072, China

## Abstract

To clarify the clinical value of serums total triiodothyronine (TT3), total thyroxine (TT4), free triiodothyronine (FT3), and free thyroxine (FT4) and provide a more eligible and economic strategy to assess thyroid function. A total of 2,673 participants (500 patients with hyperthyroidism, 500 patients with hypothyroidism, and 1,673 healthy people) were involved in our study. Serums TT3, TT4, FT3, and FT4 and thyrotropin (TSH) were measured with VIDAS fluorescent enzyme immunoassay. The Pearson correlation between TT3, TT4, FT3, and FT4 and TSH was determined to identify the most important indicator for thyroid function besides TSH. The correlation of TT4, and FT4 with TSH was statistically significant in healthy individuals (*P* < 0.01), and the *R*-values were −0.065 and −0.152, respectively. The correlation of TT4, FT4, TT3, and FT3 with TSH was statistically significant in patients with hyperthyroidism, and the *R*-values were −0.241, −0.225, −0.195, and −0.176, respectively. The correlation of TT4, FT4, TT3, and FT3 with TSH was statistically significant in patients with hypothyroidism, and the *R*-values were −0.322, −0.262, −0.179, and −0.136, respectively. In our opinion, TSH and FT4 are the most valuable indicators in assessing thyroid function in a healthy population, and TSH and TT4 are the most meaningful in hyperthyroidism and hypothyroidism.

## 1. Introduction

The thyroid is an important gland involved in the metabolism, growth, development, and maintenance of the internal environment [1, 2]. Thyroid dysfunction is very commonly encountered in clinical practice. The prevalence of thyroid dysfunction is approximately 5–10% during a lifetime [3]. In a survey from 110 countries (mostly in developing countries), hyperthyroidism and hypothyroidism were considered responsible for the morbidity at large [4]. TSH regulates the synthesis and secretion of the thyroid hormone through the hypothalamic-pituitary-thyroid axis [5] and is considered the primary indicator to assess thyroid function [6]. Currently, thyrotropin (TSH), free thyroxine (FT4), or FT4 combined with total triiodothyronine (TT3) is recommended for use as indicators in laboratory testing to assess thyroid function clinically (e.g., in the guidelines of the American Thyroid Association (ATA)) [7, 8]. However, this recommendation is not based on credible clinical evidence or on the grounds of a large enough demographic data set. According to the existing references, the clinical value of the four parameters (TT3, TT4, FT3, and FT4) has not been elaborated clearly. Therefore, in our study, based on numerous clinical data, we analyse the correlation between TT3, TT4, FT3, FT4, and TSH in healthy people and in patients with hyperthyroidism or hypothyroidism, in order to answer the above question and provide clinicians with the most meaningful and economical testing parameters to evaluate thyroid function.

## 2. Subjects and Methods

### 2.1. Study Population

A total of 2,673 participants (500 patients with hyperthyroidism, 500 patients with hypothyroidism, and 1,673 healthy people) were enrolled from Shanghai Tenth People's Hospital from Jan. 2010 to Dec. 2013. Three groups were analysed: (1) healthy population: 20–60 y, 489 males, and 1184 females; all the subjects were enrolled from the medical centre and presented with no thyroid diseases as evaluated through history, blood testing, and thyroid ultrasound; (2) hyperthyroidism (all diagnosed with Graves' disease): 20–75 y, 136 males, and 364 females; (3) hypothyroidism (all diagnosed with Hashimoto's thyroiditis): 20–75 y, 155 males, and 345 females. All patients with hyperthyroidism or hypothyroidism were from the outpatient department of endocrinology and had been confirmed to be in good general health by history, physical examination, screening blood tests, X-ray, electrocardiogram, and so forth. The clinical characteristics of the included participants are shown in Table 1.

### 2.2. Laboratory Analysis

Venous blood (2 mL) was collected from participants using a vacutainer. Serums TT3, TT4, FT3, FT4, and TSH were measured by chemiluminescent immunometric assay (Roche Diagnostics GmbH). The manufacturer's reference limits are TT3 (1.2–3.4 nmol/L), TT4 (54–174 nmol/L), FT3 (3.5–6.5 pmol/L), FT4 (10.2–31 pmol/L), and TSH (0.35–5.5 mIU/L).

### 2.3. Statistical Analysis

We used SPSS software version 15.0 for data analysis (IBM SPSS 15, Inc., Chicago, IL). The results are presented as the mean ± SD. The Pearson correlation was adopted to identify the most important indicator besides TSH, excluding the interference of aging at the same time. The correlation was considered statistically significant and more relevant when *P* < 0.05 and the absolute value of the *R*-value was close to 1.

## 3. Results

### 3.1. Baseline Characteristics

The baseline data of the individuals and concentrations of TT4, TT3, FT4, FT3, and TSH from the three groups are presented in Tables 1 and 2.

### 3.2. Correlation Analysis

The correlations between TT4, FT4, and TSH were statistically significant (*P* < 0.01) in 1673 healthy people, and the *R*-values were −0.065 and −0.152, respectively. The correlation between FT4 and TSH was the maximum. The correlations between TT4, TT3, FT4, FT3, and TSH were all statistically significant (*P* < 0.01) in 500 patients diagnosed with hyperthyroidism. The *R*-values were −0.241, −0.225, −0.195, and −0.176, respectively. The correlation between TT4 and TSH was the maximum. The correlations between TT4, FT4, TT3, FT3, and TSH were all statistically significant (*P* < 0.01) in 500 patients with hypothyroidism. The *R*-values were −0.322, −0.262, −0.179, and −0.136, respectively. The correlation between TT4 and TSH was the maximum (Table 3).

## 4. Discussion

TSH is considered the most important indicator for the evaluation of thyroid function [6]. FT3 and FT4 are the active biological state in plasma, and therefore, FT3 and FT4 are considered to be sensitive and meaningful indicators for the diagnosis of thyroid disease. However, the determination of free thyroxine is not very reliable and stable; it may be affected by several factors, such as thyroglobulin (TGB), serious illness, or certain drugs that interfere with the binding of hormones. In addition, the measuring of FT3 and FT4 is indirect, with no direct quantitative determination. Therefore, the recommendation that TSH combined with FT4 is the basis of evaluation of thyroid function is not reliable enough for both clinicians and patients. Based on this consideration, we analyse the evaluation of serums TT3, TT4, FT3, and FT4 in a Chinese population in order to provide more convenient and meaningful parameters for thyroid function.

Among the 500 patients diagnosed with hyperthyroidism, 500 patients diagnosed with hypothyroidism, and 1,673 healthy persons, we analysed the Pearson correlations for serums TT3, TT4, FT3, and FT4 with TSH. FT4 is found to be associated with TSH at the maximum level in healthy people. The correlation between TT4 and TSH in patients diagnosed with hyperthyroidism or hypothyroidism is the maximum, which suggests that FT4 and TSH are the most valuable indicators to consider in a healthy population, and TT4 and TSH are the most valuable indicators to consider in patients with hyperthyroidism or hypothyroidism.

The role of serum T3 is limited, either TT3 or FT3, because they are generally normal in patients diagnosed with hypothyroidism. This is mainly due to the increased TSH and the functional role generated by the increased conversion of type 2 iodinated thyronine deiodinase to residual thyroid tissue. Because 80% of T3 comes from deiodination of T4, the T4 levels increase and in theory T3 levels should also increase concomitantly. However, in patients diagnosed with hypothyroidism who are treated long-term with levothyroxine (L-T4), serum T3 is usually maintained at a stable level, which suggests that energy metabolism is changed by a T4 dependent pathway [9]. Therefore, T4 is more reliable than T3 in assessing thyroid function in patients with hypothyroidism. From Castellano's study, the analyses of five serum indicators, including TT4, TT3, FT4, FT3, and TSH, in patients with hypothyroidism showed that the correlation between TT4, FT4, and TSH was closer than the correlation of TT3, FT3, and TSH with TSH which was the most important indicator in the diagnosis of hypothyroidism [6]. Thus, it can be interpreted that T4 is more suitable than T3 in assessing thyroid function in hypothyroidism patients. In a case report, a 59-year-old woman diagnosed with hypothyroidism was treated with L-T4, and TSH concentrations expectedly fell three months later. However, after stopping L-T4 treatment, TSH level increased quickly but with a still higher FT4 and FT3 status [10]. This might be explained by the fact that nonspecific binding of the blood sample and assay reagent affected the measurement result of FT4 and FT3 [10]. However, the fact that serums FT4 and FT3 do not match the clinical manifestations is to some extent caused by the interference of the measurement method [11–13]. From clinical practice, the measurement of serum FT4 is an indirect assessed value and is less stable and repeatable compared with TT4. Evelin Mingote and other scholars thought that patients with elevated levels of TSH and decreased TT4 but not FT4 levels in hypothyroidism had a worse prognosis [14]. Therefore, the clinical value of TT4 may be greater than FT4 in the evaluation of thyroid function in hypothyroidism. Although the recommendation from ATA guidelines suggests TSH and FT4 for hypothyroidism patients' following measurement, we suggest that serums TSH and TT4 are more credible and dependable, at least in Chinese patients.

It is commonly known that the severity of hyperthyroidism is only partially correlated with serum FT4 and FT3 levels, and a report of 25 patients diagnosed with Graves' disease showed that there was no strong correlation between thyroid symptom spectrum and serum free thyroxine levels [15]. Studies have shown that, in patients who took thiazole zinc, serums TSH and TT4 changed significantly, while TT3 did not change significantly [16]. However, the asynchronous change of TT4 and TT3 is not well explained; therefore, it is generally agreed that TT3 does not reflect thyroid function very well. According to research based on animals, in cats suffering from hyperthyroidism, over 30% had a normal TT3, while only 10% had a normal TT4 [17, 18]. Many clinicians prefer measuring TT4 rather than TT3 because of its better diagnostic sensitivity. This might be that when production of thyroid hormone begins to increase, this leads to a compensatory decrease of T4 into T3 [19]. Therefore, measurement of serum T3 is not strongly recommended for the diagnosis of hyperthyroidism, and if T3 is determined, it should always be measured together with TT4 [17, 18]. For the patient suspected with hyperthyroidism but with normal serums T4 and T3, remeasurement of serum T4 should be made at least 1 to 2 weeks later [19]. Moreover, transportation of the blood sample can also cause large deviation of FT4 measurements. For example, when the samples are stored at 37°C for 7 days, the measured values of FT4 increase by approximately 160%, while no significant change of TT4 was detected [19]. Therefore, for the lack of stability and accuracy of current clinical FT4 measurement, TT4 is significantly better than FT4 for clinical measurement [20], and TT4 is more suitable for thyroid function assessment. According to the guidelines of ATA, measurements of TT3 and FT4 are recommended in hyperthyroidism patients. TT3 is used to exclude the T3 hyperthyroidism, and FT4 is used for clinical following. From our study, we recommended that TSH and TT4 levels be used as preferred indicators to easily assess thyroid function in Chinese patients with hyperthyroidism (if excluding T3 hyperthyroidism).

Thyroid disease can always be ruled out when the serum TSH level is normal without drug administration or in the absence of obvious hypothalamic-pituitary disease [21]. However, even in a thyroid disease-free population, Gammage found that high-normal thyroid function could have an impact on the human health, such as atrial fibrillation [22]. Heeringa et al. conducted a prospective cohort study with 1,426 subjects, which showed that people with normal thyroid function could also show the correlation between TSH, FT4 and the risk of atrial fibrillation [23]. Therefore, the thyroid function testing of healthy people is equally important. Studies in healthy people > 50 years old showed that there are a variety of progressive changes in hypothalamic-pituitary-thyroid axis function. These changes will affect the accurate assessment of thyroid function. These changes on circulating T4 and TSH are minimal, while the effect on T3 is relatively larger [24, 25]. Therefore, TSH and T4 are more stable and accurate for the evaluation of thyroid function in healthy people. At any age, TSH secretion is modulated by T4 levels, and the responsiveness of the thyroid-pituitary axis is most likely different at different ages [26–28]. Chopra found that there are various reasons for the changes of thyroid function in a thyroid disease-free population [29]. One of the factors is excessive production of cytokines, including tumour necrosis factor (TNF) and interleukin (IL), and these cytokines can reduce the secretion of TSH, TT4 and inhibit gene expression of TBG in liver. The secretion and release of thyroid hormones from the thyroid gland generally occurs at the normal levels in healthy people. In the absence of factors affecting the TBG, serum thyroid hormone levels can be maintained within a certain range. Therefore, TT4 and FT4 both may reflect thyroid function. De Alfieri et al. have shown that, in populations with normal thyroid function at baseline, increasing FT4 levels tended to predict long-term mortality [30]. Combined with our data analysis showing that the correlation between FT4 and TSH is the closest in healthy people, we may speculate that, clinically, we can easily evaluate thyroid function by measuring TSH and FT4 levels in a healthy population.

In summary, we recommend TSH and FT4 as measuring indicators to assess thyroid function in healthy people and TT4 and TSH as measuring indicators to assess hypothyroidism and hypothyroidism. We think such a permutation is an accurate, convenient, and economic recommendation for clinicians. The significance of properly assessing thyroid function is that it can effectively guide the dose adjustments of antithyroid drugs or hormone replacement therapy.

## Figures and Tables

**Table 1:** Basic information.

	Healthy population	Hyperthyroidism	Hypothyroidism	Normal range
Age (years)	50.9 ± 13.3	42.8 ± 15.2	48.6 ± 17.2	
Gender (male/female)	489/1184	136/364	155/345	
BMI (kg/m²)	25.1 ± 2.1	21.9 ± 1.4	28.3 ± 1.8	
SBP (mm Hg)	129.2 ± 18.8	126.0 ± 19.1	129.0 ± 16.8	
DBP (mm Hg)	83.1 ± 9.4	79.2 ± 9.1	81.9 ± 11.9	
Cr (*μ*mol/L)	60.2 ± 12.1	55.13 ± 9.5	57.1 ± 10.9	45–84 *μ*mol/L
BUN (mmol/L)	5.00 ± 1.4	3.75 ± 0.7	6.44 ± 0.9	2.76–8.07 mmol/L
ALT (U/L)	18.89 ± 9.7	18.61 ± 7.8	17.55 ± 8.2	<45 U/L
AST (U/L)	21.6 ± 7.5	20.4 ± 7.2	31.1 ± 8.6	<32 U/L
EKG	No obvious abnormalities			
X-ray	No obvious abnormalities			
Abdominal ultrasound	No obvious abnormalities			
History of diabetes	None			

BMI: body mass index; SBP: systolic blood pressure; DBP: diastolic blood pressure; Cr: creatinine; BUN: blood urea nitrogen; ALT: alanine aminotransferase; AST: aspartate aminotransferase; EKG: electrocardiogram.

**Table 2:** TT4, TT3, FT4, FT3, and TSH of three groups.

	TT4 (nmol/L)	TT3 (nmol/L)	FT4 (pmol/L)	FT3 (pmol/L)	TSH (mIU/L)
Healthy population	114.42 ± 15.376	1.63 ± 0.429	15.07 ± 2.528	4.43 ± 0.647	1.82 ± 0.994
Hyperthyroidism	257.83 ± 78.476	6.20 ± 3.415	51.94 ± 28.175	13.19 ± 5.530	0.02 ± 0.050
Hypothyroidism	34.66 ± 13.201	1.02 ± 0.984	7.79 ± 2.822	3.04 ± 1.571	21.99 ± 24.418
Normal range	54–174	1.2–3.4	10.2–31	3.5–6.5	0.35–5.5

**Table 3:** Correlation analysis between TT4, TT3, FT4, FT3, and TSH in three groups.

	TT4	TT3	FT4	FT3
Healthy population	*R* = −0.065 *P* < 0.05	*R* = 0.034 *P* = 0.166	*R* = −0.152 *P* < 0.05	*R* = −0.023 *P* = 0.344
Hyperthyroidism	*R* = −0.241 *P* < 0.05	*R* = −0.195 *P* < 0.05	*R* = −0.225 *P* < 0.05	*R* = −0.176 *P* < 0.05
Hypothyroidism	*R* = −0.322 *P* < 0.05	*R* = −0.179 *P* < 0.05	*R* = −0.262 *P* < 0.05	*R* = −0.136 *P* < 0.05
